# Bridging intravenous thrombolysis in patients with atrial fibrillation

**DOI:** 10.3389/fneur.2022.945338

**Published:** 2022-08-03

**Authors:** Adnan Mujanovic, Christoph C. Kurmann, Tomas Dobrocky, Marta Olivé-Gadea, Christian Maegerlein, Laurent Pierot, Vitor Mendes Pereira, Vincent Costalat, Marios Psychogios, Patrik Michel, Morin Beyeler, Eike I. Piechowiak, David J. Seiffge, Pasquale Mordasini, Marcel Arnold, Jan Gralla, Urs Fischer, Johannes Kaesmacher, Thomas R. Meinel, The BEYOND-SWIFT Investigators

**Affiliations:** ^1^University Institute of Diagnostic and Interventional Neuroradiology, University Hospital Bern Inselspital, University of Bern, Bern, Switzerland; ^2^Department of Neurology, University Hospital Bern Inselspital, University of Bern, Bern, Switzerland; ^3^Department of Neurology, Vall d'Hebron University Hospital, Barcelona, Spain; ^4^Department of Diagnostic and Interventional Neuroradiology, Klinikum rechts der Isar, Technical University Munich, Munich, Germany; ^5^Department of Neuroradiology, University Hospital Reims, Reims, France; ^6^Joint Department of Medical Imaging, Toronto Western Hospital, Toronto, ON, Canada; ^7^Department of Neuroradiology, University Hospital Montpellier, Montpellier, France; ^8^Department of Neuroradiology, Clinic of Radiology and Nuclear Medicine, University Hospital Basel, Basel, Switzerland; ^9^Department of Neurology, Lausanne University Hospital, University of Lausanne, Lausanne, Switzerland; ^10^Department of Neurology, University Hospital Basel, Basel, Switzerland

**Keywords:** atrial fibrillation, oral anticoagulation, ischemic stroke, mechanical thrombectomy, intravenous thrombolysis

## Abstract

**Background and purpose:**

40% of acute ischemic stroke patients treated by mechanical thrombectomy (MT) have a clinical history of atrial fibrillation (AF). The safety of bridging intravenous thrombolysis (IVT) (MT + IVT) is currently being discussed. We aimed to analyze the interaction between oral anticoagulation (OAC) status or AF with bridging IVT, regarding the occurrence of symptomatic intracranial hemorrhage (sICH) and functional outcome.

**Materials and Methods:**

Multicentric observational cohort study (BEYOND-SWIFT registry) of consecutive patients undergoing MT between 2010 and 2018 (*n* = 2,941). Multinomial regression models were adjusted for prespecified baseline and plausible pathophysiological covariates identified on a univariate analysis to assess the association of AF and OAC status with sICH and good outcomes (90-day modified Rankin Scale score 0–2).

**Results:**

In the total cohort (median age 74, 50.6% women), 1,347 (45.8%) patients had AF. Higher admission National Institutes of Health Stroke Scale (NIHSS) score (aOR 1.04 [95% 1.02–1.06], per point of increase) and prior medication with Vitamin K antagonists (VKA) (aOR 2.19 [95% 1.27–3.66]) were associated with sICH. Neither AF itself (aOR 0.71 [95% 0.41–1.24]) nor bridging IVT (aOR 1.08 [0.67–1.75]) were significantly associated with increased sICH. Receiving bridging IVT (aOR 1.61 [95% 1.24–2.11]) was associated with good 90-day outcome, with no interaction between AF and IVT (*p* = 0.92).

**Conclusion:**

Bridging IVT appears to be a reasonable clinical option in selected patients with AF. Given the increased sICH risk in patients with VKA, subgroup analysis of the randomized controlled trials should analyze whether patients with VKA might benefit from withholding bridging IVT.

**Registration:**

clinicaltrials.gov; Unique identifier: NCT03496064.

## Introduction

Atrial fibrillation (AF) causes ~20% of all acute ischemic stroke (AIS) cases (1), and 40% of large-vessel occlusion patients with AIS who undergo mechanical thrombectomy (MT) have a clinical history of AF (2).

In addition, AF-related stroke is usually associated with increased disability, mortality, and treatment-related costs (3, 4).

The role of intravenous thrombolysis (IVT) in the treatment of patients with large-vessel occlusion presenting directly to MT-capable centers is currently under investigation. Two recent randomized controlled trials (RCTs) have shown noninferiority of direct MT when compared with bridging IVT, which is defined as direct MT preceded by IVT (5, 6). A third trial failed to demonstrate noninferiority of direct MT (7), while the fourth trial showed that MT was neither superior nor inferior over the bridging IVT approach (8).

Recently, Akbik et al. reported that patients with AF who underwent treatment with bridging IVT were significantly more associated with an increased risk of symptomatic intracranial hemorrhage (sICH) when compared with patients with non-AF undergoing bridging IVT (9). There was also no reported benefit in 90-day functional outcome in patients with AF who have received bridging IVT (9). Therefore, arguing that patients with AF would be a subgroup that might particularly benefit from withholding IVT before MT (9). However, the conducted analysis had not accounted for the use of anticoagulants and the type of anticoagulants that patients with AF were using, which are known to be important in the context of pursuing the bridging approach (10, 11). Although “therapeutic” oral anticoagulation (OAC) formerly represented an absolute contraindication for IVT, this is rather a continuum than a dichotomized situation. Additionally, reversal agents have enabled the use of IVT even in patients with anticoagulation in the therapeutic range.

We hypothesized that OAC and different OAC types, rather than AF, influence the occurrence of sICH and functional outcomes in bridging patients. Therefore, we aimed to determine a potential interaction of IVT and AF adjusting for the use of OAC and different OAC types.

## Materials and methods

### BEYOND-SWIFT registry

The Bernese-European Registry for Ischemic Stroke Patients Treated Outside Current Guidelines With Neurothrombectomy Devices Using the Solitaire FR with the Intention for Thrombectomy (BEYOND-SWIFT) is an international, multicenter, observational registry, which evaluates patient outcomes after MT (Unique identifier: NCT03496064). Full registry information has been previously published (12). In brief, this study included patients with large vessel occlusion acute ischemic stroke, who were treated with a Medtronic market-released MT device (Solitaire) in seven comprehensive stroke centers. To increase the total sample size, additional pooling was performed from another comprehensive stroke center not originally included in the BEYOND-SWIFT registry, as this center had available all variables of interest which were included in the original registry. To avoid selection bias, the same inclusion criteria were applied for all 8 centers, amounting to a total of 2,944 included patients (13). Informed consent was obtained for patients unless the institutional board waived the need to do so. An overview of included patients is available in Supplementary Table 1. The local ethics committee approved data pooling and analysis (Kantonale Ethikkommission Bern, ID: 2018-00766). Study data are available from the corresponding author on reasonable request and after clearance by the local ethics committee.

### Baseline characteristics

Patient data included demographic characteristics (i.e., age and sex), clinical presentation, and laboratory values at admission to the treating institution [blood pressure, blood glucose levels, international normalized ratio (INR), platelet count, stroke severity quantified on the National Institute of Health Stroke Scale (NIHSS)], medical history (pre-stroke independence defined as modified Rankin Scale (mRS) score 0–2, diabetes, arterial hypertension, dyslipidemia, smoking, history of stroke, and AF), and pre-stroke medication (anticoagulation, antiplatelet and statin). Anticoagulation status was defined as a current prescription for Vitamin-K antagonist (VKA), direct oral anticoagulants (DOAC), or not taking OAC. Diagnosis of AF included both known and newly diagnosed paroxysmal and persistent AF identified by electrocardiography and/or 24 h ECG monitoring. Due to the absolute contraindication of IVT and OAC, we assume that all included patients with AF who underwent bridging IVT had subtherapeutic OAC levels, or received reversal agents prior to IVT application.

### Outcome of interest

The primary aim of this analysis was to assess the interaction between AF and bridging IVT on the rates of sICH and good outcomes at 3 months, adjusting for the type of OAC status. Definition from the European Co-Operative Acute Stroke Study-II was used to describe sICH as any hemorrhagic transformation and worsening by equal to or >4 on the NIHSS (14). mRS score at 90 days after the indexed event was used for functional outcome assessment, where mRS score 0–2 was defined as a good outcome.

### Statistical analysis

Results are reported as “median [interquartile range (IQR)]” and “*n* (%)” unless specified otherwise. Fisher exact has been used for categorical and Mann-Whitney *U* for continuous variables. Logistic regression results are displayed as odds ratios (OR) for simple regression or adjusted OR (aOR) for multinomial regression analyses, with their corresponding 95% confidence intervals (CIs), where aORs of the independent variables were plotted as forest plots. Regression was adjusted for prespecified baseline and pathophysiologically plausible covariates identified on univariate analysis, which could influence the following outcomes: age (continuous variable), sex (binary variable), NIHSS on admission (continuous variable, aOR referring to one point increase), diabetes (binary variable), hypertension (binary variable), dyslipidemia (binary variable), smoking (binary variable), IVT usage (binary variable), AF (binary variable), and OAC status (0 = None, 1 = VKA, and 2 = DOAC). The interaction term AF^*^IVT was included in the model as well. A sensitivity analysis on the association of INR in patients with VKA, with an admission INR of <1.7, was performed to test for a dose-dependent association of INR with sICH. For this analysis, INR was included in the sICH model. All tests are 2-sided, with a significance level set at α = 0.05. Presented analyses were conducted using R version 4.0.0 (15).

## Results

Our final study population included 2,941 patients, 50.6% of women, with a median age of 74 years (IQR 62 – 82). In this analysis, 1,347 (45.8%) patients had comorbid AF. Patients with AF were more likely to be women, older, have higher pre-stroke dependence, higher admission NIHSS, more likely to have OAC prescribed and statin medication, more likely to have diabetes, hypertension, and previous ischemic stroke, and have higher admission glucose and INR values and lower admission platelet count (Supplementary Table 2). Notably, 16.1% of patients had preceding anticoagulation (4.0% DOAC, 12.1% VKA).

Out of all patients with AF, 46.9% underwent bridging IVT. Those receiving bridging IVT were more likely to be male, be transferred from a referring center, have better pre-stroke independence, less likely to use OAC and statin medication, less likely to have hypertension or previous stroke, and had higher admission glucose and lower admission INR levels (Table 1). On admission, patients underwent either a CT or an MRI scan (74.5 vs. 25.5%).

**Table 1:** Patients with atrial fibrillation stratified by the use of intravenous thrombolysis.

**Variable**		**Missing *n* (%)**	**Overall**	**AF without IVT**	**AF with IVT**	***p***
*N* (%)			1,347	715 (53.1)	632 (46.9)	
Age on admission (median [IQR])			78 [69, 84]	78 [70, 84]	77 [68, 83]	0.213
Sex (Female %)			761 (56.5)	423 (59.2)	338 (53.5)	0.041
Type of admission (Direct %)		2 (0.1)	772 (57.4)	438 (61.4)	334 (52.8)	0.002
Admission imaging	CT	10 (0.8)	995 (74.5)	522 (73.5)	473 (75.6)	0.430
	MRI		341 (25.5)	188 (26.5)	153 (24.4)	
Pre-stroke independence (mRS score ≤ 2, %)		185 (13.7)	1,023 (88.0)	545 (86.0)	478 (90.5)	0.022
NIHSS on admission (median [IQR])		16 (1.2)	16 (11,20)	17 (11,20)	16 (11,20)	0.21
Anticoagulation (%)	None	61 (4.5)	944 (73.4)	399 (58.2)	545 (90.7)	<0.001
	DOAC		67 (5.2)	61 (8.9)	6 (1.0)	
	VKA		275 (21.4)	225 (32.8)	50 (8.3)	
Antiplatelet (%)	None	55 (4.1)	869 (67.3)	480 (69.6)	389 (64.6)	0.001
	Mono		400 (31.0)	191 (27.7)	209 (34.7)	
	Double		23 (1.8)	19 (2.8)	4 (0.7)	
Statins (Yes %)		145 (10.8)	386 (32.1)	232 (35.9)	154 (27.7)	0.003
Diabetes (Yes %)		10 (0.7)	285 (21.3)	154 (21.7)	131 (20.9)	0.795
Hypertension (Yes %)		7 (0.5)	989 (73.8)	548 (77.0)	441 (70.2)	0.006
Dyslipidemia (Yes %)		14 (1)	618 (46.4)	330 (46.6)	288 (46.1)	0.89
Smoking (Yes %)		55 (4.1)	223 (17.3)	112 (16.2)	111 (18.5)	0.294
Previous stroke (Yes %)		238 (17.7)	174 (15.7)	118 (19.3)	56 (11.2)	<0.001
Systolic blood pressure on admission (mmHg) (median [IQR])		358 (26.6)	150 [133, 167]	151 [132, 170]	150 [133, 164]	0.235
Diastolic blood pressure on admission (mmHg) (median [IQR])		361 (26.8)	81 [70, 94]	82 [70, 95]	80 [70, 93]	0.731
Glucose on admission (mmol/L) (median [IQR])		335 (24.9)	7.4 [6.1, 10.7]	7.2 [6.1, 9.6]	7.8 [6.2, 14.8]	0.003
INR on admission (median [IQR])		437 (32.4)	1.06 [1, 1.2]	1.1 [1, 1.3]	1.03 [1, 1.1]	<0.001
Platelet count on admission (median [IQR])		379 (28.1)	212 [170, 264]	211 [170, 270]	215 [171, 261]	0.62

AF, atrial fibrillation; IVT, intravenous thrombolysis; mRS, modified Rankin Scale; NIHSS, National Institutes of Health Stroke Scale; DOAC, direct oral anticoagulants; VKA, vitamin K antagonists; INR, international normalized ratio; ASPECTS, Alberta Stroke Program Early CT score.

Rates of sICH did not differ between patients with AF receiving and not receiving IVT (5.2% vs. 5.6%), but patients with AF receiving IVT had significantly better 90-day outcomes (*p* < 0.001, Table 2). Patients who underwent their 90-day follow-up examination after the indexed event had generally better baseline factors when compared to patients who did not show up to their follow-up 90-day examination (Supplementary Table 3).

**Table 2:** Outcome characteristics of patients with atrial fibrillation stratified by the use of intravenous thrombolysis.

**Variable**	**Missing n (%)**	**Overall**	**AF without IVT**	**AF with IVT**	***p***
N (%)		1,347	715 (53.1)	632 (46.9)	
sICH (%)	15 (1.1)	72 (5.4)	37 (5.2)	35 (5.6)	0.852
mRS score 0–2 at 3 months (%)	287 (21.3)	418 (39.4)	182 (33.3)	236 (46.0)	<0.001
Mortality at 3 months (%)	526 (39)	206 (25.1)	124 (28.1)	82 (21.6)	0.038

AF, atrial fibrillation; IVT, intravenous thrombolysis; sICH, symptomatic intracranial hemorrhage; mRS, Modified Rankin Scale.

On an unadjusted analysis of all patients, the occurrence of sICH was significantly associated with higher NIHSS (OR 1.04 [95% 1.02–1.06]) and preceding VKA therapy (OR 1.58 [95% 1.00–2.43], Supplementary Table 4), while there was no significant association in other predictors. sICH rates stratified by different OAC categories were 5.5 vs. 7.8 vs. 5.1% for DOAC, VKA, and no patients with OAC, respectively (*p* = 0.119).

**Figure 1:**
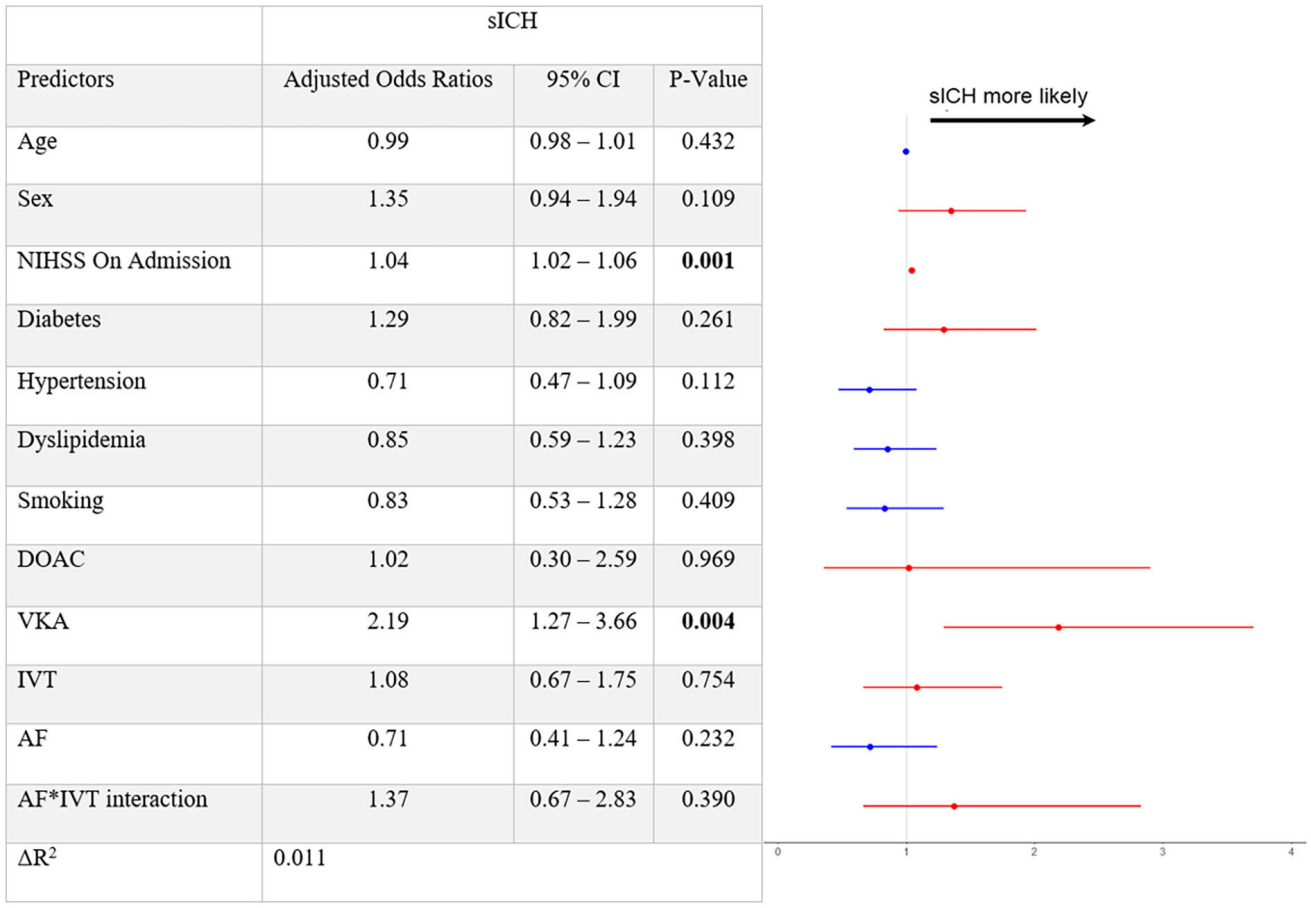
Multivariable logistic regression model with symptomatic intracranial hemorrhage defined as a dependent variable. sICH, symptomatic intracranial hemorrhage; CI, confidence interval; NIHSS, National Institutes of Health Stroke Scale; DOAC, direct oral anticoagulants; VKA, vitamin-K antagonists; IVT, intravenous thrombolysis; AF, atrial fibrillation. After adjusting for confounders, the fitted multivariable logistic regression model for symptomatic intracranial hemorrhage (sICH) reveals the following significant associations: admission NIHSS (aOR 1.04 [95% 1.02–1.06], per point of increase) and VKA (aOR 2.19 [95% 1.27–3.66], not using OAC was used as a reference variable for VKA and DOAC usage). IVT (aOR 1.08 [95% 0.67–1.75]) and AF (aOR 0.71 [95% 0.41–1.24]) are not associated with sICH, and neither was the AF*IVT interaction term.

Multivariate logistic regression analysis revealed higher NIHSS on admission (aOR 1.04 [95% 1.02–1.06], per point of increase) and using VKA (aOR 2.19 [95% 1.27–3.66]) to be significantly associated with increasing sICH rates. Conversely, neither AF (aOR 0.71 [95% 0.41–1.24]) nor IVT (aOR 1.08 [0.67–1.75]) were significantly associated with an increased risk of sICH (Figure 1). There was also no significant interaction between the AF^*^IVT term and sICH (*p* = 0.39). Even when the analysis was not adjusted for OACs, again neither AF (aOR 0.91 [95% 0.55–1.50]), nor IVT (aOR 1.08 [95% 0.68–1.72]), nor the AF^*^IVT interaction (*p* = 0.72) was significantly associated with increased sICH risk (Supplementary Table 5). Sensitivity analysis restricted only to patients with VKA revealed that increasing INR doses with a cutoff value of 1.6 were not associated with increased sICH rates (Supplementary Table 6).

**Figure 2:**
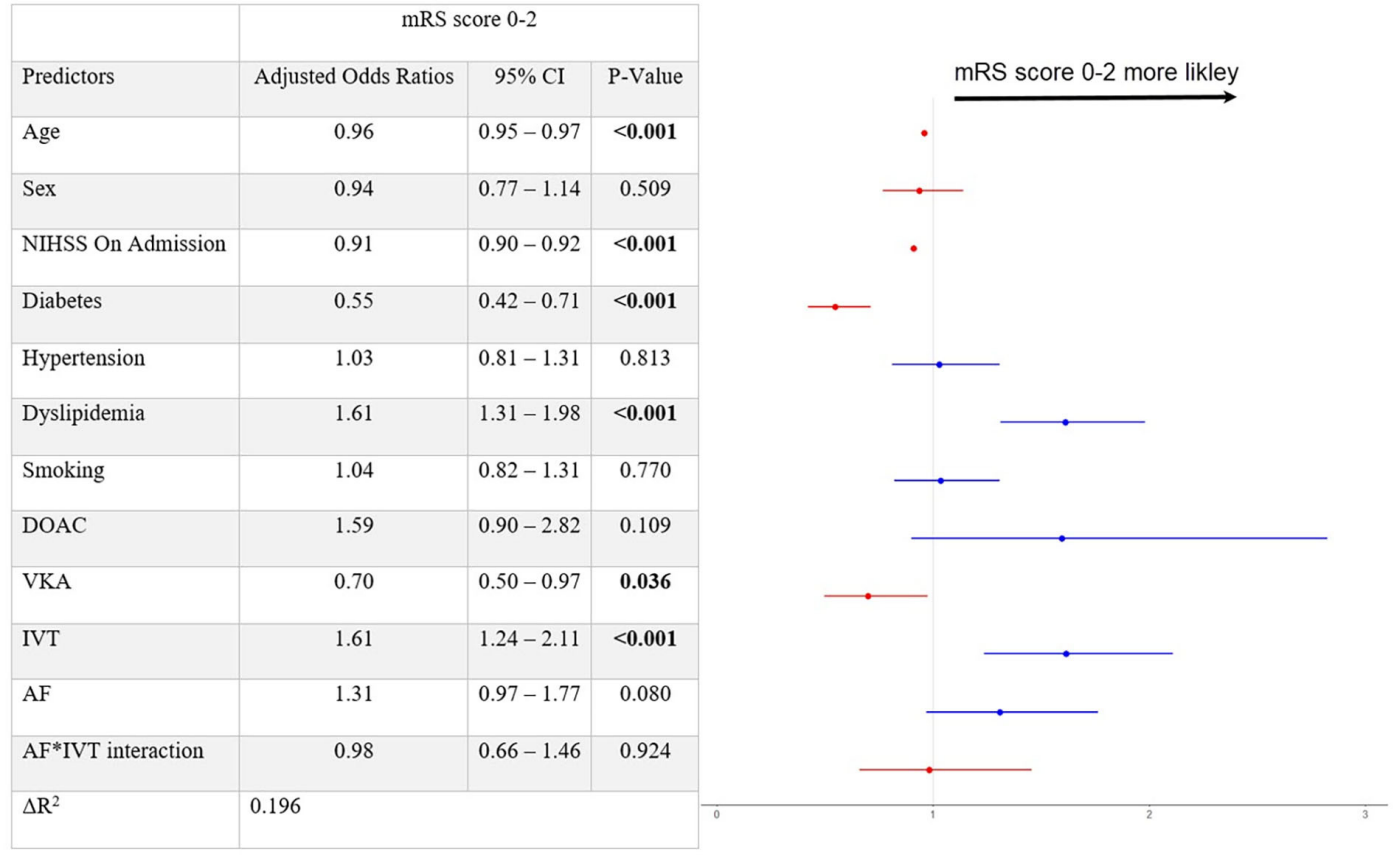
Output of the logistic regression model with the favorable patient outcome at 3 months as a dependent variable. mRS, Modified Rankin Scale; CI, confidence interval; NIHSS, National Institutes of Health Stroke Scale; DOAC, direct oral anticoagulants; VKA, vitamin-K antagonists; sICH, symptomatic intracranial hemorrhage; IVT, intravenous thrombolysis; AF, atrial fibrillation. After adjusting for confounders, fitted multivariable logistic regression model for favorable patient outcome at 3 months (mRS score 0–2) reveals significant association for IVT [aOR 1.61 (95% 1.24–2.11)].

For good outcome at 3 months, having dyslipidemia (aOR 1.61 [95% 1.31–1.98]) and receiving IVT (aOR 1.61 [95% 1.24–2.11]) was observed as significant, while, again, no significant interaction was found for the AF^*^IVT term (*p* = 0.92, Figure 2). Ordinal regression analysis supported the association of IVT and lower 3-month mRS rates (aOR 0.74 [95% 0.58–0.93], Supplementary Table 7). Comparable results for main outcomes of interest were also shown when excluding patients enrolled from the center who was not originally in the BEYOND-SWIFT registry (Supplementary Table 8), or when adding the final thrombolysis in cerebral infarction (TICI) score in the regression models (Supplementary Table 9).

## Discussion

This study on the use of bridging IVT has the following main findings: (1) bridging IVT was associated with good functional outcomes in patients with AF, and no interaction of AF and IVT regarding sICH was found. (2) VKA, but not DOAC, therapy was associated with increased rates of sICH.

In our study, almost half (46%) of the patients undergoing MT had concomitant AF, similar to other studies (2), stressing that this is a very frequent clinical scenario. Obviously, patients with AF are likely to receive OAC, if AF was known before stroke. In our cohort, 16.1% of patients had preceding OAC (4% DOAC and 12.1% VKA). Therefore, we aimed to disentangle the relation between AF, OAC, and the potential risks and benefits of the bridging approach.

In their recent analysis, Akbik et al. reported an association between bridging IVT and increased sICH rates in patients with AF, without any benefit in functional outcome. However, their report did not account for the use and type of OAC. Our analysis shows that the type of OAC is very likely to determine the influence of sICH risk in this patient subgroup. This does not exclude an option that the findings of Akbik et al. might have been by chance, further requiring the findings of this group to be replicated by other study groups.

Although the point estimate indicated an increased risk of sICH with bridging IVT as one would expect, from the IVT RCT (5–8), no interaction of AF and IVT regarding sICH was found. Therefore, we presume that AF alone should not be a reason to withhold bridging IVT in patients otherwise qualifying for it. Before deciding on whenever to give or withhold IVT, only patient characteristics on the initial presentation are available. Therefore, any post-interventional parameters (e.g., the TICI score) were voided from the main analyses as they do not play a role in the decision-making process at the time point of deciding on the IVT treatment.

Previous research from BEYOND-SWIFT restricted only to patients with low Alberta Stroke Program Early Computed Tomography score showed excess sICH risk in patients undergoing the bridging approach, although these analyses did not explore the presence of AF, nor the relationship between OAC and IVT (16). In the present study, we have found an increased risk of sICH in patients with VKA, whereas no such association could be found for patients with DOAC or patients with AF themselves. This is in line with preliminary data showing no increased sICH risk in DOAC- (17), but an increased risk of sICH in VKA-patients (18). Importantly, the results of this study are drawn under the assumption that all patients with OAC had subtherapeutic levels at the time point of IVT administration. This assumption is based on the current guidelines of the American Heart Association and American Stroke Association where IVT is contraindicated in patients with OAC: with DOAC being classified as a relative contraindication for IVT unless the time since the last intake is >48 h (19). For patients with VKA, with an INR of 1.6 or below, IVT can be offered (19). However, IVT might be considered in individual patients with therapeutic OAC levels or recent DOAC ingestion based on expert opinions (11, 20).

Our data show similar sICH rates when comparing patients with AF being treated with and without IVT (5.6 vs. 5.2%, respectively). Even though the association of increasing INR and sICH rates has been previously discussed, with a 2-fold increase in sICH risk for every 1 unit rise in INR (21), we could not replicate this in our patient subanalysis with VKA-only. Mortality at 3 months was significantly lower in patients with AF who have received IVT, although missing values in this category were copious due to loss to follow-up. Even so, the reported overall mortality rate falls within the range of other studies on MT, as reported by a recent meta-analysis of ten MT RCTs (8.6–30.1%) (22).

An editorial article comparing arguments on direct MT vs. bridging IVT in patients with IVT-eligible concluded that the bridging approach will most likely remain the standard of care for the majority of patients with large vessel occlusion strokes (23). Newest guidelines from the European Stroke Organization and European Society of Minimally Invasive Neurological Therapy corroborated this, with strong recommendations for the bridging IVT approach over MT alone (24). Future individual patient data meta-analysis of RCTs on bridging IVT should analyze whether patients with VKA might be a subgroup that potentially benefits from proceeding with direct MT, ideally taking into consideration the last time point of OAC intake and drug-plasma levels. Until further evidence from RCTs becomes available, we suggest not to skip IVT in patients with AF who otherwise qualify for bridging IVT.

### Limitations

This is a retrospective registry analysis limiting the generalizability of our results to other cohorts. There was a notable percentage of missing outcome variables due to transfer patients who were lost to follow-up. Patients presenting at 90-day follow-up tended to do better at baseline when compared with those lost to follow-up, prompting possible systemic bias. We did not assess relevant time metrics for endovascular treatment, such as door-to-needle or door-to-groin-puncture time, even though these might influence patient outcomes. Although we adjusted for measured factors in the models, treatment selection and unmeasured confounding may bias outcomes comparisons. The registry did not assess compliance, adherence, and anticoagulant drug plasma levels, which could have resulted in a subtherapeutic concentration of medications in some patients and would have further impacted the patient outcome (25). Data on IVT selection modality for patients on anticoagulants were not captured in this registry.

### Conclusion

After adjusting for current OAC status and different OAC types, bridging IVT did not show a significant association with increased sICH rates. Moreover, bridging IVT appears to promote good functional outcomes at 3 months in patients with AF when compared with MT alone. In the context of AF, the bridging approach appears to be a reasonable clinical option in selected patients being admitted to experienced high-volume thrombectomy centers. Given the increased sICH risk in patients with VKA, subgroup analysis of the RCT should analyze whether patients with VKA might benefit from withholding bridging IVT.

## Data availability statement

Study data are available from the corresponding author upon reasonable request and after clearance by the local ethics committee.

## Ethics statement

The studies involving human participants were reviewed and approved by Kantonale Ethikkommission Bern, ID: 2018-00766. The patients/participants provided their written informed consent to participate in this study.

## Author contributions

AM: conceptualization, data curation, formal analysis, investigation, methodology, and writing of the original draft. CK: data curation, formal analysis, methodology, and writing of the original draft. TD: data curation, formal analysis, and reviewing and editing of the final version. MO-G: data curation, investigation, and project administration. CM: data curation, investigation, and reviewing and editing of the final version. LP and VM: data curation and reviewing and editing of the final version. VC: data curation, validation, and reviewing and editing of the final version. MP, PMi, MB, EP, DS, PMo, and MA: validation and reviewing and editing of the final version. JG: resources, validation, and reviewing and editing of the final version. UF and JK: conceptualization, supervision, and reviewing and editing of the final version. TM: conceptualization, supervision, writing of the original draft, and reviewing and editing of the final version. All authors contributed to the article and approved the submitted version.

## Funding

This study was funded by the Bangerter-Rhyner Foundation and the Swiss Academy of Medical Sciences. Open access funding provided by University of Bern.

## Conflict of interest

LP reports personal fees from Balt, Phenox, and Microvention outside the submitted work. VM reports personal fees from Medtronic and Stryker during the conduct of this study. PMo reports research support from Siemens, Cerenovus, iSchemaview, Medtronic, and Stryker and is receipt of honoraria and consultation fees from Medtronic, Cerenovus, Phenox, and Microvention. MA reports personal fees from Bayer, Bristol-Myers Squibb, Medtronic, Amgen, Daiichi Sankyo, Nestlé Health Sciences, Boehringer Ingelheim, and Covidien during the conduct of this study. JG is the global coprincipal investigator of the SWIFT DIRECT trial (Solitaire with the Intention for Thrombectomy Plus Intravenous tPA Versus DIRECT Solitaire Stent-Retriever Thrombectomy in Acute Anterior Circulation Stroke; Medtronic), therefore, consultant Medtronic. He receives Swiss National Science Foundation grants for magnetic resonance imaging in stroke. UF reports grants from Medtronic during the conduct of this study; grants from Medtronic; and others from Medtronic, Stryker, and CSL Behring outside the submitted work and his board membership at the Journal of NeuroInterventional Surgery. JK reports grants from the Swiss Academy of Medical Sciences/Bangerter Foundation, Swiss Stroke Society, and Clinical Trial Unit Bern during the conduct of this study. TM reports research support from the Bangerter Rhyner Foundation, the Swiss National Foundation, and the Swiss Heart Foundation.

The remaining authors declare that the research was conducted in the absence of any commercial or financial relationships that could be construed as a potential conflict of interest.

## Publisher's note

All claims expressed in this article are solely those of the authors and do not necessarily represent those of their affiliated organizations, or those of the publisher, the editors and the reviewers. Any product that may be evaluated in this article, or claim that may be made by its manufacturer, is not guaranteed or endorsed by the publisher.

## Abbreviations

AF: atrial fibrillation
MT: mechanical thrombectomy
IVT: intravenous thrombolysis
OAC: oral anticoagulants
VKA: vitamin K antagonists
DOAC: direct oral anticoagulants
sICH: symptomatic intracranial hemorrhage
INR: international normalized ratio
RCT: randomized controlled trials.
