# Supplementary material for: Bridging intravenous thrombolysis in patients with atrial fibrillation
Source: Front Neurol. 2022 Aug 3;13:945338. doi: 10.3389/fneur.2022.945338 (PMC9382124; doi:10.3389/fneur.2022.945338)
Supplement: Supplementary file 1 [file Data_Sheet_1.pdf]

Supplementary Material

1 Table of Contents

- List of all BEYOND-SWIFT Investigators
- Supplementary Table 1 BEYOND-SWIFT Registry Overview
- Supplementary Table 2 Cohort Characteristics Stratified by Diagnosis of Atrial Fibrillation
- Supplementary Table 3 Baseline Factors of Patients Who Have and Did Not Have Follow-Up Examination Three Months After the Indexed Event
- Supplementary Table 4 Unadjusted Analysis With Symptomatic Intracranial Hemorrhage as the Outcome Variable
- Supplementary Table 5 Analysis With Symptomatic Intracranial Hemorrhage as the Outcome Variable Unadjusted for Oral Anticoagulants
- Supplementary Table 6 Sensitivity Analysis of International Normalized Ratio on Outcome of Patients Under Vitamin-K Antagonist Therapy
- Supplementary Table 7 Ordinal Regression With Modified Rankin Scale Score at 3 months as the Outcome Variable
- Supplementary Table 8 Main Outcomes of Interest With Excluded Patients From One Center Which Was Not Part of the BEYOND-SWIFT Registry
- Supplementary Table 9 Regression Models for Main Outcomes of Interest With Added TICI Score

2 List of all BEYOND-SWIFT Investigators

BEYOND-SWIFT (Bernese-European Registry for Ischemic Stroke Patients Treated Outside Current Guidelines With Neurothrombectomy Devices Using the SOLITAIRE FR With the Intention for Thrombectomy) investigators: Leonidas Panos, MD, Department of Neurology, University Hospital Bern, Inselspital, University of Bern, Bern, Switzerland; Panagiotis Chaloulos-Iakovidis, MD, Department of Neurology, University Hospital Bern, Inselspital, University of Bern, Bern, Switzerland; Alex Brehm, MD, Department of Neuroradiology, Clinic of Radiology & Nuclear Medicine, University Hospital Basel, Basel, Switzerland; Marc Ribo, MD, and Manuel Requena, MD, Department of Neurology, Vall d'Hebron University Hospital, Barcelona, Spain; Steven D. Hajdu, MD, Department of Radiology, CHUV Lausanne, Laussane, Switzerland; Amel Benali, MSc, Department of Neuroradiology, CHU Montpellier, Montpellier, France; Benjamin Friedrich, MD, Department of Diagnostic and Interventional Neuroradiology, Klinikum rechts der Isar, Technical University Munich, Munich, Germany; Joanna Schaafsma, MD, Department of Neurology, Toronto Western Hospital, Ontario, Canada; Matthias Gawlitza, MD, Department of Neuroradiology, CHU Reims, Reims, France; Jan Liman, MD, Department of Neurology, University Medical Center Gottingen, University of Gottingen, Gottingen, Germany.

3 Supplementary Table 1 BEYOND-SWIFT Registry Overview

**Center**	N	Time period	LVO anterior circulation (ICA, M1, M2)	Lost to follow- up (mRS day 90)	ASPECTS available (LVO anterior circulation)	% MRI as initial imaging modality	ASPECTS grading	Admission and 24h NIHSS	mRS at 90 days	TICI grading	EC approval	Responsible EC
Inselspital Bern, University Hospital Bern, University of Bern, Bern, Switzerland	1317	2010-2018	90.8% (1195/1317)	6.1% (80/1317)	97.7% (1168/1195)	48.5% (637/1317)	Research fellow blinded to clinical data	Board certified stroke neurologists	Stroke neurologists on scheduled clinical visits. Structured telephone interviews if the patient was unable to attend (either by physician or mRS certified stroke nurse).	Operator-measured	Yes	Kantonale Ethik Kommission Bern
Toronto Western Hospital - University Health Network, University of Toronto, Toronto, Canada	60	2014-2017	88.3% (53/60)	0% (0/60)	53/53	1.7% (1/60)	Prospective, by neuroradiologist	Board certified stroke neurologists	Clinical visits at the university hospital. For patients still in rehabilitation facilities, a mRS certified nurse schedules telephone interviews.	Operator-measured	Yes	IRB Toronto
Klinikum rechts der Isar, Technical University Munich, Munich, Germany	206	2009-2017	74.3% (153/206)	18.4% (38/206)	151/153	2.4% (5/206)	Rerospective by neuroradiologist	Board certified stroke neurologists	mRS was evaluated either by face-to-face assessments (by stroke neurologists) or standardized telephone interviews (by certified study nurses).	Operator Measured	Yes	Ethikkommission der medizinischen Fakultät der Technischen Universität München
University Hospital Vall d'Hebron, Barcelona, Spain	418	2010-2017	85.7% (359/419)	20.0% (84/419)	319/360	0% (0/491)	Prospective, by neurologist/neuroradiologist on call	Board certified stroke neurologists	Stroke neurologists on scheduled clinical visits. Structured telephone interviews if unable to attend.	Operator Measured	Yes	CEIC H. Vall d'Hebrond
CHUV, Lausanne University Hospital, Lausanne, Switzerland	139	2012-2017	124/139 (89.2%)	26.6% (37/139)	113/124	0.1% (1/139)	Consensus stroke neurologist and neuroradiologist (not blinded)	Board certified stroke neurologists	mRs was assessed by Rankin-certified physicians at 3 months in the outpatient clinic, or alternatively through a structured telephone interview by Rankin-certified personnel.	Operator-measured	Yes	Commission Ethique de Recherche, Canton de Vaud
Montpellier CHU, University Hospital Montpellier, Montpellier, France	149	2015-2017	97.3% (145/149)	4.0% (6/149)	109/145	82.1% (96/117)	Operator-measured	Board certified stroke neurologists	Stroke neurologists on scheduled clinical visits. Structured telephone interviews if unable to attend.	Operator-measured	(Yes)	Consent was waived owning to the retrospective design (favorable opinion by CNIL)
CHU Reims, University Hospital Reims, Reims, France	108	2013 - 2017	90.7% (98/108)	0% (0/108)	96/98	92.6% (100/108)	Retrospective, certified neuroradiologist	Board certified stroke neurologists	Stroke physician on clinical visits at university hospital or remote outpatient center.	Retrospective, certified neuroradiologist	(Yes)	Consent was waived owning to the retrospective design
**Non-BEYOND center in this publication**												
University Hospital Göttingen, Göttingen, Germany	547	2015-2019	88.7% (477/547)	15.4% (84/547)	89.1% (425/477)	0% (0/547)	Retrospective certified neuroradiologist	Board certified stroke neurologists	Stroke physician on clinical visits at university hospital or telephone interview	Retrospective, certified neuroradiologist	Yes	Ethik Kommission (UMG)
**Total**	**2944**											

Adapted from Kaesmacher et al with permission. Copyright © 2020, The Authors (CC-BY).

4 **Supplementary Table 2 Cohort and Outcome Characteristics Stratified by Diagnosis of Atrial Fibrillation.**

Variable		Missing n(%)	Overall	Without AF	With AF	p
N (%)			2,941	1,594 (54.2)	1,347 (45.8)	
Age on admission (median [IQR])			74 [62, 82]	70 [58, 79]	78 [69, 84]	<0.001
Sex (Female %)			1487 (50.6)	726 (45.5)	761 (56.5)	<0.001
Type of admission (Direct %)		3 (0.1)	1714 (58.3)	942 (59.1)	772 (57.4)	0.361
Pre-stroke independence (mRS ≤ 2)		348 (11.8)	2341 (90.3)	1318 (92.1)	1023 (88.0)	0.001
NIHSS on admission (median [IQR])		46 (1.6)	16 [10, 20]	15 [9, 19]	16 [11, 20]	<0.001
Anticoagulation (%)	None	156 (5.3)	2336 (83.9)	1392 (92.9)	944 (73.4)	<0.001
	DOAC		111 (4.0)	44 (2.9)	67 (5.2)	
	VKA		338 (12.1)	63 (4.2)	275 (21.4)	
Antiplatelet (%)	None	150 (5.1)	1909 (68.4)	1040 (9.4)	869 (67.3)	0.486
	Mono		834 (29.9)	434 (29.0)	400 (31.0)	
	Dual		48 (1.7)	25 (1.7)	23 (1.8)	
Statins (Yes %)		362 (12.3)	777 (30.1)	391 (28.4)	386 (32.1)	0.044
Diabetes (Yes %)		44 (1.5)	566 (19.5)	281 (18.0)	285 (21.3)	0.029
Hypertension (Yes %)		42 (1.4)	1997 (68.9)	1008 (64.7)	989 (73.8)	<0.001
Dyslipidemia (Yes %)		59 (2)	1416 (49.1)	798 (51.5)	618 (46.4)	0.006
Smoking (Yes %)		131 (4.5)	702 (25.0)	479 (31.6)	223 (17.3)	<0.001
Previous stroke (Yes %)		464 (15.8)	344 (13.9)	170 (12.4)	174 (15.7)	0.018
Systolic blood pressure on admission (mmHg) (median [IQR])		779 (26.5)	150 [131, 168]	150 [130, 168]	150 [133, 167]	0.464
Diastolic blood pressure on admission (mmHg) (median [IQR])		786 (26.7)	80 [70, 92]	80 [70, 90]	81 [70, 94]	0.001
Glucose on admission (mmol/L) (median [IQR])		727 (24.7)	7.1 [6.0, 10.3]	7 [5.9, 10.1]	7.4 [6.1, 10.7]	<0.001
INR on admission (median [IQR])		968 (32.9)	1.02 [1, 1.1]	1 [1, 1.1]	1.06 [1, 1.2]	<0.001
Platelet count on admission (median [IQR])		823 (28)	219 [176, 267]	223 [182, 269]	212 [171, 264]	0.007
IVT (Yes %)			1432 (48.7)	800 (50.3)	632 (46.9)	0.075
Final TICI score	0 – 2a		476 (16.2)	192 (14.3)	284 (17.9)	0.032
	2b - 3		2452 (83.7)	1152 (85.7)	1300 (82.0)	
OUTCOME						
sICH (Yes %)		31 (1.1)	163 (5.6)	91 (5.8)	72 (5.4)	0.733
mRS score 0-2 at 3 months (Yes %)		652 (22.2)	968 (42.3)	550 (44.8)	418 (39.4)	0.012
Mortality (Yes %)		1113 (37.8)	462 (25.3)	256 (25.4)	206 (25.1)	0.914

AF: Atrial Fibrillation; NIHSS: National Institutes of Health Stroke Scale; DOAC: Direct Oral Anticoagulants; VKA: Vitamin-K Antagonists; INR: International Normalized Ratio; IVT: Intravenous Thrombolysis; TICI: Thrombolysis in Cerebral Infarction; sICH: symptomatic intracranial hemorrhage; mRS: modified Rankin Scale.

5 **Supplementary Table 3 Baseline Factors of Patients Who Have and Did Not Have Follow-Up Examination Three Months After the Indexed Event**

			90-Day Mortality				90-Day mRS 0-2			
**Variable**		**Missing n (%)**	**Overall**	**Have Follow-Up**	**Do Not Have Follow-Up**	**p**	**Overall**	**Have Follow-Up**	**Do Not Have Follow-Up**	**p**
N			1,347	821	526		1,347	1,060	287	
Age on admission (median [IQR])			78 [69, 84]	77.36 [68, 83]	79 [70, 85]	0.01	78 [69, 84]	78 [69, 84]	79 [70, 85]	0.107
Sex (Female %)			761 (56.5)	450 (54.8)	311 (59.1)	0.133	761 (56.5)	600 (56.6)	161 (56.1)	0.931
NIHSS on admission (median [IQR])		16 (1.2)	16 [11, 20]	17 [12, 21]	15 [10, 20]	0.001	16 [11, 20]	17 [11, 20]	16 [10, 20]	0.118
Anticoagulation (%)	None	61 (4.5)	944 (73.4)	575 (75.0)	369 (71.1)	0.002	944 (73.4)	746 (74.4)	198 (70.0)	0.302
	DOAC		67 (5.2)	49 (6.4)	18 (3.5)		67 (5.2)	49 (4.9)	18 (6.4)	
	VKA		275 (21.4)	143 (18.6)	132 (25.4)		275 (21.4)	208 (20.7)	67 (23.7)	
Antiplatelet (%)	None	55 (4.1)	869 (67.3)	513 (66.7)	356 (68.1)	0.087	869 (67.3)	669 (66.5)	200 (69.9)	0.126
	Mono		400 (31.0)	247 (32.1)	153 (29.3)		400 (31.0)	322 (32.0)	78 (27.3)	
	Double		23 (1.8)	9 (1.2)	14 (2.7)		23 (1.8)	15 (1.5)	8 (2.8)	
Statin (Yes %)		145 (10.8)	386 (32.1)	221 (32.4)	165 (31.8)	0.884	386 (32.1)	300 (32.6)	86 (30.5)	0.554
Diabetes (Yes %)		10 (0.7)	285 (21.3)	145 (17.8)	140 (26.9)	<0.001	285 (21.3)	226 (21.5)	59 (20.7)	0.838
Hypertension (Yes %)		7 (0.5)	989 (73.8)	579 (70.9)	410 (78.4)	0.003	989 (73.8)	781 (74.0)	208 (73.2)	0.866
Dyslipidemia (Yes %)		14 (1)	618 (46.4)	383 (47.1)	235 (45.3)	0.564	618 (46.4)	493 (47.0)	125 (44.0)	0.408
Smoking (Yes %)		55 (4.1)	223 (17.3)	155 (19.9)	68 (13.2)	0.002	223 (17.3)	180 (17.8)	43 (15.4)	0.388
Previous stroke (Yes %)		238 (17.7)	174 (15.7)	115 (14.1)	59 (20.3)	0.002	174 (15.7)	126 (14.9)	48 (18.4)	0.258
Systolic blood pressure on admission (mmHg) (median [IQR])		358 (26.6)	150 [133, 167]	150 [133, 167]	150 [133, 168.]	0.865	150 [133, 167]	150 [133, 167]	150 [134, 168]	0.57
Diastolic blood pressure on admission (mmHg) (median [IQR])		361 (26.8)	81 [70, 94]	81 [70, 93]	80 [70, 95]	0.637	81 [70, 94]	80 [70, 93]	83 [70, 95]	0.264
Glucose on admission (mmol/L) (median [IQR])		335 (24.9)	7.40 [6.11, 10.67]	6.80 [5.90, 8.30]	12.90 [6.80, 124.00]	<0.001	7.40 [6.11, 10.67]	7.50 [6.20, 11.70]	7.10 [6.00, 9.00]	0.014
INR on admission (median [IQR])		437 (32.4)	1.06 [1.00, 1.20]	1.06 [1.00, 1.19]	1.08 [1.00, 1.20]	0.842	1.06 [1.00, 1.20]	1.06 [1.00, 1.20]	1.07 [1.00, 1.17]	0.133
Platelet count on admission (median [IQR])		379 (28.1)	212 [171, 264]	206 [167, 254]	224 [176, 280]	<0.001	212 [171, 264]	210 [168, 262]	224 [178, 279]	0.027

mRS: modified Rankin Scale; NIHSS*:* National Institutes of Health Stroke Scale; DOAC: Direct Oral Anticoagulants; VKA: Vitamin-K Antagonists; INR: International Normalized Ratio.

6 Supplementary Table 4 Unadjusted Analysis With Symptomatic Intracranial Hemorrhage as the Outcome Variable

Variable	sICH	
	OR [95% CI]	P-value
Age	0.99 [0.98 – 1.00]	0.167
Sex (Male)	1.14 [0.83 – 1.57]	0.409
NIHSS on admission	1.04 [1.02 – 1.06]	**<0.001**
Diabetes	1.23 [0.83 – 1.78]	0.289
Hypertension	0.82 [0.59 – 1.14]	0.233
Dyslipidemia	0.79 [0.57 – 1.10]	0.17
Smoking	0.96 [0.65 – 1.41]	0.863
DOAC	1.08 [0.42 – 2.32]	0.852
VKA	1.58 [1.00 – 2.43]	**0.041**
IVT	1.19 [0.86 – 1.64]	0.281
AF	0.93 [0.67 – 1.28]	0.673

sICH: symptomatic intracranial hemorrhage; OR: Odds Ratios; CI: confidence interval; NIHSS: National Institutes of Health Stroke Scale; DOAC: Direct Oral Anticoagulants; VKA: Vitamin K Antagonists; IVT: Intravenous Thrombolysis; AF: Atrial Fibrillation.

7 Supplementary Table 5 Analysis With Symptomatic Intracranial Hemorrhage as the Outcome Variable Unadjusted for Oral Anticoagulants

	sICH		
Predictor	Adjusted Odds Ratios	95% CI	P-Value
Age	1.00	0.98 – 1.01	0.513
Sex	1.24	0.87 – 1.75	0.233
NIHSS On Admission	1.04	1.02 – 1.07	**<0.001**
Diabetes	1.32	0.85 – 2.00	0.207
Hypertension	0.75	0.50 – 1.12	0.157
Dyslipidemia	0.79	0.55 – 1.13	0.197
Smoking	0.86	0.55 – 1.29	0.473
IVT	1.08	0.68 – 1.72	0.736
AF	0.91	0.55 – 1.50	0.704
AF*IVT interaction	1.13	0.57 – 2.25	0.716
ΔR^2	0.01		

sICH: symptomatic intracranial hemorrhage; CI: confidence interval; NIHSS: National Institutes of Health Stroke Scale; IVT: Intravenous Thrombolysis; AF: Atrial Fibrillation.

8 Supplementary Table 6 Sensitivity Analysis of International Normalized Ratio on Outcome of Patients Under Vitamin-K Antagonist Therapy

	sICH		
Predictor	Adjusted Odds Ratios	95% CI	P-Value
Age	1.05	0.97 – 1.16	0.284
Sex	0.47	0.03 – 4.88	0.547
NIHSS On Admission	1.12	0.97 – 1.31	0.128
Diabetes	0.43	0.02 – 3.65	0.489
Hypertension	0.29	0.02 – 7.64	0.376
Dyslipidemia	1.21	0.14 – 12.98	0.860
Smoking	27.12	2.15 – 667.52	**0.017**
INR per 0.1 increase (INR up to, and with, 1.6 included in this analysis)	1.28	0.71 – 2.49	0.414
ΔR^2	0.11		

sICH: Symptomatic Intracranial Hemorrhage; CI: confidence interval; NIHSS: National Institutes of Health Stroke Scale; INR: International Normalized Ratio.

9 **Supplementary Table 7 Ordinal Regression With Modified Rankin Scale Score at 3 months as the Outcome Variable**

	Modified Rankin Scale Score at 3 months		
Predictor	Adjusted Odds Ratios	95% CI	P-Value
Age	1.04	1.03 – 1.05	**<0.001**
Sex	1.06	0.85 – 1.32	0.617
NIHSS On Admission	1.09	1.08 – 1.11	**<0.001**
Diabetes	1.68	1.28 – 2.22	**<0.001**
Hypertension	0.94	0.72 – 1.24	0.676
Dyslipidemia	0.74	0.59 – 0.93	**0.008**
Smoking	1.17	0.87 – 1.59	0.292
DOAC	0.66	0.39 – 1.09	0.109
VKA	1.22	0.91 – 1.64	0.175
IVT	0.74	0.58 – 0.93	**0.011**

CI: confidence interval; NIHSS: National Institutes of Health Stroke Scale; DOAC: Direct Oral Anticoagulants; VKA: Vitamin K Antagonists; IVT: Intravenous Thrombolysis.

10 Supplementary Table 8 Main Outcomes of Interest With Excluded Patients From One Center Which Was Not Part of the BEYOND-SWIFT Registry

	sICH			mRS 0-2 at 90-Days		
**Predictors**	Adjusted Odds Ratios	95% CI	P-Value	Adjusted Odds Ratios	95% CI	P-Value
**Age**	1.00	0.98 – 1.01	0.727	0.96	0.95 – 0.97	**<0.001**
**Sex (Male)**	1.47	1.00 – 2.20	0.053	0.93	0.75 – 1.16	0.530
**NIHSS on Admission**	1.04	1.01 – 1.06	**0.004**	0.91	0.90 – 0.93	**<0.001**
**Diabetes**	1.43	0.86 – 2.31	0.150	0.56	0.41 – 0.76	**<0.001**
**Hypertension**	0.72	0.46 – 1.12	0.138	1.34	1.03 – 1.75	**0.030**
**Dyslipidemia**	0.79	0.53 – 1.17	0.242	1.48	1.18 – 1.86	**0.001**
**Smoking**	0.83	0.52 – 1.31	0.436	1.08	0.84 – 1.39	0.542
**DOAC**	1.00	0.30 – 2.57	0.993	1.41	0.79 – 2.50	0.240
**VKA**	2.45	1.35 – 4.29	**0.002**	0.69	0.47 – 1.00	**0.050**
**IVT**	1.26	0.76 – 2.11	0.368	1.46	1.09 – 1.95	**0.012**
**AF**	0.70	0.38 – 1.26	0.239	1.33	0.96 – 1.85	0.086
**AF*IVT interaction**	1.32	0.60 – 2.89	0.488	1.07	0.69 – 1.67	0.756
**ΔR^2**	0.01			0.18		

sICH: symptomatic intracranial hemorrhage; mRS: Modified Rankin Scale; CI: confidence interval; NIHSS: National Institutes of Health Stroke Scale; ; DOAC: Direct Oral Anticoagulants; VKA: Vitamin K Antagonists; IVT: Intravenous Thrombolysis; AF: Atrial Fibrillation.

11 **Supplementary Table 9 Regression Models for Main Outcomes of Interest With Added TICI Score**

	sICH			mRS 0-2		
**Predictors**	Adjusted Odds Ratios	95% CI	P-Value	Adjusted Odds Ratios	95% CI	P-Value
**Age**	1.00	0.98 – 1.01	0.700	0.96	0.95 – 0.97	**<0.001**
**Sex (Male)**	1.55	1.04 – 2.31	**0.031**	0.88	0.70 – 1.11	0.282
**NIHSS on admission**	1.04	1.01 – 1.06	**0.006**	0.91	0.90 – 0.93	**<0.001**
**Diabetes**	1.37	0.82 – 2.21	0.211	0.58	0.42 – 0.79	**0.001**
**Hypertension**	0.71	0.46 – 1.12	0.135	1.35	1.04 – 1.78	**0.027**
**Dyslipidemia**	0.81	0.54 – 1.21	0.298	1.50	1.19 – 1.90	**0.001**
**Smoking**	0.85	0.53 – 1.34	0.501	1.06	0.82 – 1.38	0.637
**DOAC**	1.08	0.32 – 2.77	0.889	1.25	0.70 – 2.24	0.451
**VKA**	2.30	1.27 – 4.03	**0.005**	0.71	0.47 – 1.05	0.087
**TICI 2b-3**	0.44	0.29 – 0.69	**<0.001**	3.93	2.79 – 5.62	**<0.001**
**IVT**	1.27	0.76 – 2.14	0.352	1.47	1.09 – 1.98	**0.013**
**AF**	0.75	0.41 – 1.35	0.340	1.29	0.92 – 1.80	0.141
**AF*IVT interaction**	1.31	0.60 – 2.87	0.504	1.06	0.67 – 1.66	0.813
**ΔR^2**	0.02			0.23		

sICH: symptomatic intracranial hemorrhage; mRS: Modified Rankin Scale; CI: confidence interval; NIHSS: National Institutes of Health Stroke Scale; ; DOAC: Direct Oral Anticoagulants; VKA: Vitamin K Antagonists; TICI: Thrombolysis in Cerebral Infarction; IVT: Intravenous Thrombolysis; AF: Atrial Fibrillation.
